# Association between delayed outbreak identification and SARS-CoV-2 infection and mortality among long-term care home residents, Ontario, Canada, March to November 2020: a cohort study

**DOI:** 10.2807/1560-7917.ES.2024.29.41.2300719

**Published:** 2024-10-10

**Authors:** Kevin A Brown, Sarah A Buchan, Adrienne K Chan, Andrew Costa, Nick Daneman, Gary Garber, Michael Hillmer, Aaron Jones, James M Johnson, Dylan Kain, Kamil Malikov, Richard G Mather, Allison McGeer, Kevin L Schwartz, Nathan M Stall, Jennie Johnstone

**Affiliations:** 1 Public Health Ontario, Toronto, Canada; 2 Dalla Lana School of Public Health, University of Toronto, Toronto, Canada; 3 The Institute for Health Policy, Management, and Evaluation, University of Toronto, Toronto, Canada; 4 Division of Infectious Diseases, Sunnybrook Research Institute, Toronto, Canada; 5 Department of Medicine, University of Toronto, Toronto, Canada; 6 McMaster University, Hamilton, Canada; 7 School of Epidemiology and Public Health, University of Ottawa, Ottawa, Canada; 8 Ontario Ministry of Health, Toronto, Canada; 9 Department of Family Medicine, Queen’s University, Kingston, Canada; 10 Sinai Health, Toronto, Canada; 11 Department of Laboratory Medicine and Pathobiology, University of Toronto, Toronto, Canada; 12 St. Joseph’s Health System, Toronto, Canada; 13 Women’s College Hospital, Toronto, Canada

**Keywords:** long term care facility, LTCF, nursing homes, aged care facilities, outbreak, timeliness, SARS-CoV-2, infection prevention and control

## Abstract

**Background:**

Late outbreak identification is a common risk factor mentioned in case reports of large respiratory infection outbreaks in long-term care (LTC) homes.

**Aim:**

To systematically measure the association between late SARS-CoV-2 outbreak identification and secondary SARS-CoV-2 infection and mortality in residents of LTC homes.

**Methods:**

We studied SARS-CoV-2 outbreaks across LTC homes in Ontario, Canada from March to November 2020, before the COVID-19 vaccine rollout. Our exposure (late outbreak identification) was based on cumulative infection pressure (the number of infectious resident-days) on the outbreak identification date (early: ≤ 2 infectious resident-days, late: ≥ 3 infectious resident-days), where the infectious window was −2 to +8 days around onset. Our outcome consisted of 30-day incidence of secondary infection and mortality, based on the proportion of at-risk residents with a laboratory-confirmed SARS-CoV-2 infection with onset within 30 days of the outbreak identification date.

**Results:**

We identified 632 SARS-CoV-2 outbreaks across 623 LTC homes. Of these, 36.4% (230/632) outbreaks were identified late. Outbreaks identified late had more secondary infections (10.3%; 4,437/42,953) and higher mortality (3.2%; 1,374/42,953) compared with outbreaks identified early (infections: 3.3%; 2,015/61,714; p < 0.001, mortality: 0.9%; 579/61,714; p < 0.001). After adjustment for 12 LTC home covariates, the incidence of secondary infections in outbreaks identified late was 2.90-fold larger than that of outbreaks identified early (OR: 2.90; 95% CI: 2.04–4.13).

**Conclusions:**

The timeliness of outbreak identification could be used to predict the trajectory of an outbreak, plan outbreak measures and retrospectively provide feedback for quality improvement, with the objective of reducing the impacts of respiratory infections in LTC home residents.

**Key public health message**

**What did you want to address in this study and why?**

We aimed to measure the association between how late a SARS-CoV-2 outbreak is identified and the final size of the outbreak in long-term care homes. Such an indicator of the outbreak identification delay could help gauge the resources needed to control a SARS-CoV-2 outbreak and make comparisons across long-term care homes for quality improvement purposes.

**What** **have we learnt from this study?**

We classified outbreaks based on the cumulative number of infectious resident-days elapsed on the date the outbreak was identified, i.e. late ≥ 3 resident-days vs early ≤ 2 days. Across 632 outbreaks recorded in Ontario, Canada from March to November 2020, those identified late had a substantially higher incidence of secondary infections (10%) compared to outbreaks identified early (3%). Mortality was also higher in outbreaks identified late (3%) vs early (1%).

**What are the implications of your findings for public health?**

Outbreaks identified late evolved to be larger and more severe. Measurement of outbreak identification delays, leveraging the concept of resident infection pressure, could be used to plan outbreak responses and guide quality improvement initiatives.

## Introduction

Following the emergence of severe acute respiratory syndrome coronavirus 2 (SARS-CoV-2) in early 2020, many countries faced frequent and large SARS-CoV-2 outbreaks in long-term care (LTC) homes; when LTC home residents were infected, advanced age and comorbidity burden drove high case fatality rates [1,2]. The result was a disproportionate burden of mortality among LTC home residents, which early in the pandemic represented 30–80% of deaths across European and North American countries, although LTC home residents represented less than 1% of these countries’ respective populations [3-5]. 

Several factors have been identified that affect SARS-CoV-2 introductions and transmission within LTC homes. Community transmission is known to be associated with the probability of introduction of SARS-CoV-2 into LTC homes by staff [6], and factors including for-profit status [7], LTC home quality rating [8] and shared multi-bedded rooms [9] have been shown to drive the size of outbreaks after introduction. Case identification delays are also known to be an important driver of SARS-CoV-2 transmission within LTC homes [10]. Research from 2020, early in the COVID-19 pandemic, identified a high incidence of infection among residents and staff already at the time of the first identified case (outbreak identification) [11]. Early outbreak identification can enable prompt implementation of measures to contain transmission, particularly in more crowded LTC homes where transmission can occur more rapidly [12]. In Ontario, Canada, access to timely testing in the general population and in LTC homes improved substantially over the course of 2020 with the addition of staff SARS-CoV-2 testing policies; data reflected this with higher testing rates and lower test positivity in fall of 2020 compared to spring of 2020 [13].

To our knowledge, the association between delays in SARS-CoV-2 outbreak identification and the eventual size of LTC home outbreaks has not been examined empirically to date. Using granular resident and staff information on SARS-CoV-2 infection outbreaks during the pandemic period before the rollout of SARS-CoV-2 vaccines in Ontario, Canada [14], we examined how delays in LTC home SARS-CoV-2 outbreak identification were associated with the subsequent incidence of infection and mortality.

## Methods

### Study population

We conducted a retrospective cohort study of SARS-CoV-2 outbreaks across 623 LTC homes in Ontario, Canada from 1 March 2020 to 14 November 2020, a period ending 1 month before the COVID-19 vaccine rollout in Ontario LTC homes. Homes could be included more than once if they experienced multiple outbreaks. Laboratory-confirmed LTC home resident and staff (including family caregivers) SARS-CoV-2 cases were included. SARS-CoV-2 testing in Ontario during this period was primarily based on nasopharyngeal swab specimens that were analysed using nucleic acid amplification.

### Data sources

Data for all detected SARS-CoV-2-infected residents and staff related to LTC home outbreaks were obtained from Ontario’s Case and Contact Management (CCM) database. For each case, CCM included information on date of symptom onset (when symptomatic), specimen collection date and positive test result date. Monthly facility-level information on resident characteristics were obtained from the Continuing Care Reporting System, which is based on quarterly Resident Assessment Instrument Minimum Dataset (RAI-MDS) resident assessments [15]; information on occupancy, facility ownership and bed types were obtained from the Ontario Ministry of Long-Term Care inspections branch [9]. Datasets were cleaned and merged based on the facility name or identifier, using R version 4.0.4.

### Outbreak definition

**Figure 1:**
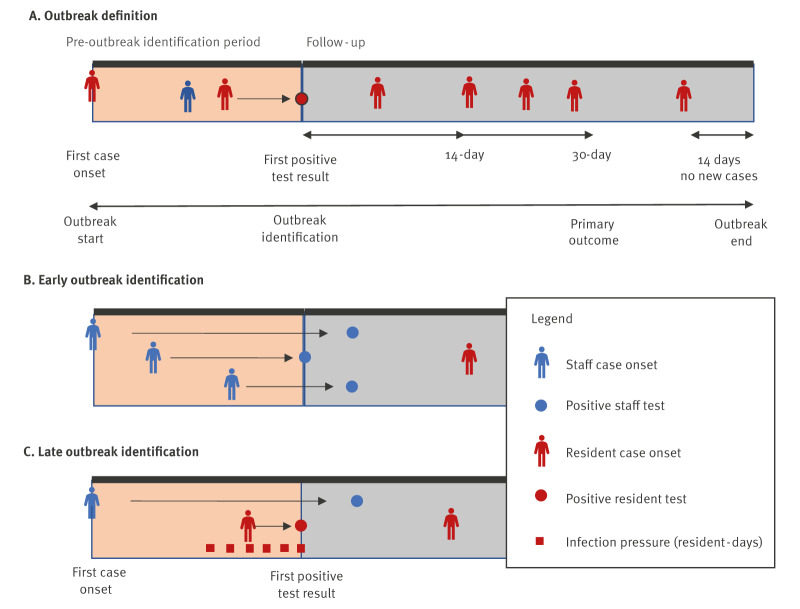
Definitions used to define timeliness of SARS-CoV-2 outbreak identification in long-term care homes, Ontario, Canada, 1 March–14 November 2020

A distinct LTC home outbreak (Figure 1) was defined as an occurrence of one resident or staff case with onset at least 14 days since the last case (based on Ontario SARS-CoV-2 outbreak surveillance definitions [16]). Onset date was defined as the first of symptom onset date or specimen collection date, in order to capture the first evidence of SARS-CoV-2 infection for each case. All subsequent resident and staff cases were included in the outbreak until 14 days passed with no new cases with an onset. Outbreaks could include a single case, as per provincial outbreak definitions. For each outbreak, we defined the outbreak identification date as the first positive test result date, and based on this, we defined a pre-outbreak identification period and a follow-up period (Figure 1). The pre-outbreak identification period was the time between the first resident or staff onset date up to and including the outbreak identification date. The full follow-up period for each outbreak was the period from the day after the outbreak identification date until 14 days after the last onset date of the outbreak.

ID: identification.

Panel A shows an overview of outbreak definitions, including the pre-outbreak identification period and the follow-up period. The pre-outbreak identification period starts at the time of onset of the first identified case (resident or staff) and ends on the outbreak identification date (first positive test result date, resident or staff). The follow-up period starts on the day after the pre-outbreak identification period ends and continues for 30 days (primary outcome) or for 14 days (secondary outcome) or until the end of the outbreak, 14 days after the last case (resident or staff; secondary outcome). Only resident cases in the follow-up period are counted as part of the outcome.

Panel B shows an example of an outbreak identified early (≤ 2 days of infection pressure). This outbreak has been identified before there are any resident cases; as such there are 0 days of resident infection pressure at the time of outbreak identification.

Panel C shows an example of an outbreak identified late (≥ 3 days of infection pressure). In this example, the single resident case had symptoms that started 3 days before the outbreak was identified (based on their own positive test); as such, there were already 6 days of resident infection pressure at the time of outbreak identification.

### Exposures – outbreak infection pressure and late outbreak identification

Outbreak infection pressure was defined as the number of infectious resident-days occurring in the window from 14 days prior (upper limit on one incubation period) to the outbreak identification date (Figure 1). We considered residents to be infectious for 10 days (from 2 days before onset to 8 days after), and stopped being infectious after that time, or earlier if they were hospitalised or died [12]. This definition measures the number of infectious resident-days of exposure occurring before the outbreak is identified, and relates to fundamental exposure measures in hospital-acquired infection epidemiology (colonisation pressure [17]) and invasion ecology (propagule pressure [18]). We truncated infection pressure at 25 infectious resident-days (the 98^th^ percentile). Note that this measure excludes staff infection pressure, because of the known role of staff in propagating LTC outbreaks [2]; outbreaks without staff infection pressure are likely to represent outbreaks with poor staff case identification.

We categorised outbreaks based on infection pressure as being identified early (≤ 2 infectious resident-days) or late (≥ 3 infectious resident-days), as this distinguished an outbreak identified before vs on or after, respectively, the onset date of a single resident case. Because, by definition, staff person-days of infection pressure did not contribute to the measure; an outbreak could have 0 resident-days of infection pressure if the outbreak was first identified among staff more than 2 days prior to any resident cases (Figure 1B).

### Outcomes

The primary outcome of this analysis was the 30-day secondary infection incidence, defined as the proportion of at-risk LTC home residents with onset within the first 30 days of the follow-up period. The number of at-risk residents was calculated as the occupancy of the LTC home in the month before the outbreak identification date, minus the number of LTC home residents infected in the pre-outbreak identification period. In addition, we examined two secondary follow-up windows: the infection incidence over the entire duration of the follow-up period and the 14-day infection incidence. For each of these three follow-up windows, we also examined the incidence of secondary mortality, which were SARS-CoV-2 deaths among those infected, for a total of six outcome measures.

### Covariates

Resident characteristics included: the percentage of residents aged ≥ 85 years, the percentage of female residents, the percentage of residents with university education, the percentage of residents with dementia, as well as the mean number of comorbidities including congestive heart failure, chronic obstructive pulmonary disease (COPD), cancer, diabetes and renal failure, and average activities of daily living (ADL) impairment scale. Long-term care home structural characteristics included: size (< 100 or ≥ 100 beds), profit status (municipal, non-profit or for-profit) and the home crowding index (< 2 or ≥ 2 residents per room) [9]. The crowding index was derived based on an algorithm using LTC home bed license types, and captures the average number of LTC home residents per bedroom and bathroom [9]. Other characteristics included the number of outbreaks before the outbreak identification date (0, 1, ≥ 2), the outbreak identification date (Wave 1: 1 Mar–31 Aug 2020; Wave 2: 1 Sep–14 Nov 2020) and the community incidence of SARS-CoV-2 excluding LTC home and congregate setting outbreaks in the public health region (n = 32) in the month of the outbreak per 10,000 population, based on CCM data.

### Statistical methods

To determine whether late outbreak identification and infection pressure were associated with secondary incidence of infection and mortality, logistic regression models were fitted to measure odds ratios (OR). All models were fitted using generalised additive modelling framework (mgcv package in R [19]). The outcome was specified as a quasibinomial count [20], with random intercepts accounting for facility-level clustering. Unadjusted models included only the exposure variable. Adjusted models included an additional 12 covariates: LTC home proportion of residents aged ≥ 85 years, proportion with female sex, proportion with university education, proportion with dementia, average number of comorbidities, average ADL impairment scale, logarithm of the size of the LTC home, profit status, crowding index > 2, number of prior outbreaks, public health unit community SARS-CoV-2 incidence and outbreak identification date. Outbreak identification date was included as a penalised spline with a knot for each 4-week period (n = 9 knots in main analysis). All unadjusted and adjusted models were fit once for the primary exposure, late outbreak identification, and once for the secondary exposure, outbreak infection pressure.

### Additional analyses

Regression models were repeated to further evaluate the impact of late SARS-CoV-2 outbreak identification on secondary cases and deaths, when we restricted the analysis to: (i) outbreaks in the first SARS-CoV-2 pandemic wave only (1 Mar–31 Aug 2020), (ii) outbreaks in the second SARS-CoV-2 pandemic wave only (1 Sep–14 Nov 2020) and (iii) second or subsequent outbreaks. We also conducted population simulations to estimate the number of cases and deaths that could have been averted if all outbreaks identified late were instead identified early [21]. To ensure comprehensiveness, this analysis was based on the secondary cases and secondary deaths outcome that covered the entire outbreak follow-up period. This analysis was repeated for the first and second waves separately.

## Results

Between 1 March 2020 and 14 November 2020, we identified 632 LTC home SARS-CoV-2 outbreaks, of which 412 (65.2%) occurred in Wave 1 and 220 (34.8%) occurred in Wave 2. These outbreaks occurred in 349 (56.0%) of Ontario’s 623 LTC homes. Across the 632 outbreaks (Table 1), there were 104,667 residents at risk of infection at the time of outbreak identification. The incidence of infection in residents at 30-days follow-up was 6.2% (n = 6,452), while the incidence of SARS-CoV-2 mortality was 1.9% (n = 1,953). The incidence of infection at 14 days was 3.5% (n = 3,643) while the incidence up to the end of the outbreak period was 7.6% (n = 7,904).

**Table 1:** Characteristics of long-term care home outbreaks identified early versus late, Ontario, Canada, 1 March–14 November 2020 (n = 632 outbreaks)

Variables	Overall outbreaks n = 632		Outbreak identification							p value^e^
			Early (≤ 2 days of infection pressure^a^) n = 402				Late (≥ 3 days of infection pressure^a^) n = 230			
	n	%	n		%		n		%	
Resident characteristics, mean (IDR)^b^										
Per cent residents aged ≥ 85 years	51.2 (35.8–64.3)		50.9 (35.3–64.3)				51.9 (37.5–65.1)			0.29
Per cent female	68.5 (58.1–77.1)		68.3 (58.2–77.0)				69.0 (58.0–77.3)			0.27
Per cent with university education	7.2 (1.3–14.8)		7.2 (1.3–14.9)				7.2 (1.4–14.5)			0.96
Per cent with dementia	59.8 (45.8–72.6)		60.3 (46.5–73.3)				58.9 (45.0–71.4)			0.12
Number of comorbidities	0.41 (0.31–0.52)		0.41 (0.31–0.51)				0.41 (0.30–0.53)			0.78
ADL impairment scale	3.93 (3.50–4.33)		3.92 (3.51–4.33)				3.95 (3.50–4.36)			0.28
LTC home structural characteristics										
Number of beds, mean (IDR)	166 (68–254)		163 (71–252)				172 (66–262)			0.76
Profit status										
Municipal	361	57.1	235	58.5		126		54.8		0.09
Private for-profit	170	26.9	100	24.9		70		30.4		
Private non-profit	101	16.0	67	16.7		34		14.8		
Crowding index > 2	263	41.6	161	40.0		102		44.3		0.29
Prior outbreaks										
0	349	55.2	204	50.7		145		63.0		0.09
1	183	29.0	127	31.6		56		24.3		
≥ 2	100	15.8	71	17.7		29		12.6		
Outbreak identification date										
Wave 1 (1 Mar–31 Aug 2020)	412	65.2	241		60.0		171		74.3	0.001
Wave 2 (1 Sep–14 Nov 2020)	220	34.8	161		40.0		59		25.7	
SARS-CoV-2 incidence on week of outbreak identification (cases per 100,000), mean (IDR)^c^	97.8 (16.6–232.9)		103.2 (18.2–232.9)				88.4 (15.3–236.7)			0.16
Outcomes^d^										
Secondary SARS-CoV-2 infections										
30-day follow-up	6,452	6.2	2,015	3.3		4,437		10.3		< 0.001
14-day follow-up	3,643	3.5	840	1.4		2,803		6.5		< 0.001
Full follow-up	7,904	7.6	2,893	4.7		5,011		11.7		< 0.001
Secondary SARS-CoV-2 mortality										
30-day follow-up	1,953	1.9	579	0.9		1,374		3.2		< 0.001
14-day follow-up	1,155	1.1	214	0.3		941		2.2		< 0.001
Full follow-up	2,293	2.2	793	1.3		1,500		3.5		< 0.001

ADL: activities of daily living; IDR: interdecile range (10–90^th^ percentile); LTC: long-term care.

^a^ Infection pressure is equal to the number of infectious resident-days at the time of outbreak identification, where residents were considered infectious from 2 days before onset to 8 days after onset.

^b^ Count of congestive heart failure, chronic obstructive pulmonary disease, cancer, diabetes and renal failure.

^c^ Health region community incidence excludes long-term care home and congregate care settings.

^d^ Denominator for overall (n = 104,667), early (n = 61,714), late (n = 42,953). Full follow-up defined as time from outbreak identification date until 14 days after the last detected case.

^e^ P values were based on Wald tests. For all variables except the outcomes, Wald tests were measured within a logistic regression model comparing outbreaks identified early versus late as the binary outcome, and using the variable as the only continuous or categorical predictor variable. For outcomes, these were measured within logistic count regression models with the count of cases and non-cases as the outcome and outbreak identification as the binary predictor variable.

### Late outbreak identification

Among the 632 outbreaks, 36.4% (n = 230) were identified late, after 3 or more infectious resident-days. On average, outbreaks identified late had 11.0 infectious resident-days at the time of identification (interdecile range (IDR): 3.5–20.0). There was a substantial improvement in outbreak identification between the first and second wave. Compared with outbreaks identified early, outbreaks identified late were more likely to have occurred in the first COVID-19 wave in Ontario (late: 171/230, 74.3% vs early: 241/402, 60.0%; p < 0.001).

### Association between late outbreak identification and outbreak incidence

At 30-days follow-up, outbreaks identified late had an incidence of 10.3% (4,437/42,953), compared with 3.3% (2,015/61,714) among outbreaks identified early (p < 0.001). Patterns were consistent when examining shorter (14-day follow-up: late 6.5% vs early 1.4%; p < 0.001) and longer follow-up windows (full follow-up: late 11.7% vs early 4.7%; p < 0.001) and when examining SARS-CoV-2-associated deaths (30-day follow-up: late 3.2% vs early 0.9%; p < 0.001).

**Figure 2:**
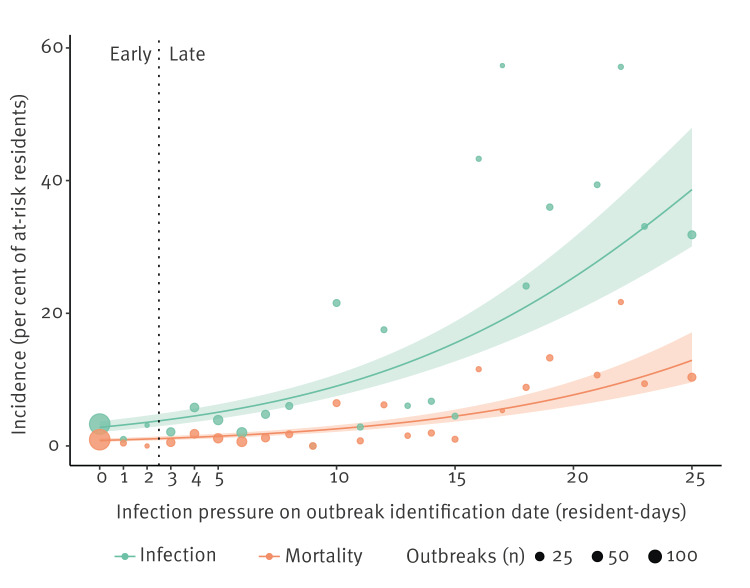
Association between infection pressure on outbreak identification date and incidence of secondary SARS-CoV-2 infection and mortality, Ontario, Canada, 1 March–14 November 2020 (n = 632 outbreaks)

At 30-days follow-up, unadjusted incidence was 3.41 times higher for outbreaks with late identification (Table 2; unadjusted OR: 3.41; 95% confidence interval (CI): 2.24–5.19). Each additional resident-day of outbreak infection pressure was associated with a 1.13-fold (unadjusted OR: 1.13; 95% CI: 1.11–1.16) increase in the odds of infection among at-risk residents in the LTC home (Figure 2). Adjustment for 12 covariates led to moderately attenuated estimates of the impact of late identification (adjusted OR: 2.90; 95% CI: 2.04–4.13) and the impact of each additional day of delay (adjusted OR: 1.10; 95% CI: 1.08–1.12). Associations were similar for the 30-day SARS-CoV-2 mortality outcome (adjusted OR: 2.47; 95% CI: 1.77–3.46). Associations were stronger when examining incidence in the shorter 14-day follow-up window (adjusted OR: 4.47; 95% CI: 2.98–6.70), compared with the entire follow-up window (adjusted OR: 2.21; 95% CI: 1.55–3.16). Coefficient estimates for the adjustment covariates for incidence of infection and mortality at 30 days are provided in the Supplementary Materials.

**Table 2:** Association between late vs early outbreak identification and SARS-CoV-2 secondary infections and mortality in long-term care home residents, Ontario, Canada, 1 March–14 November 2020 (n = 632 outbreaks)

Variables	Secondary infection incidence				Secondary mortality incidence			
	Unadjusted		Adjusted^a^		Unadjusted		Adjusted^a^	
	OR	95% CI	OR	95% CI	OR	95% CI	OR	95% CI
**30-day follow-up**								
Late (vs early)	3.41	2.24–5.19	2.90	2.04–4.13	3.49	2.26–5.39	2.47	1.77–3.46
Infection pressure^b^	1.13	1.11–1.16	1.10	1.08–1.12	1.12	1.10–1.14	1.07	1.06–1.09
**14-day follow-up **								
Late (vs early)	5.06	3.04–8.42	4.47	2.98–6.70	6.44	3.92–10.57	4.81	3.12–7.43
Infection pressure^b^	1.14	1.12–1.17	1.11	1.09–1.13	1.14	1.12–1.17	1.10	1.08–1.12
**Full follow-up** ^c^								
Late (vs early)	2.68	1.79–4.02	2.21	1.55–3.16	2.78	1.84–4.20	1.96	1.42–2.72
Infection pressure^b^	1.12	1.10–1.15	1.09	1.07–1.11	1.11	1.09–1.13	1.06	1.05–1.08

CI: confidence interval; LTC: long-term care; OR: odds ratio.

^a^ Adjusted models included the following 12 covariates: beds in the LTC home, LTC home proportion of residents aged ≥ 85 years, proportion of females, proportion with university education, proportion with dementia, average number of comorbidities, average activities of daily living (ADL) impairment scale, crowding index > 2, profit status, prior outbreaks, outbreak identification date (spline with one knot for each month) and public health unit community SARS-CoV-2 incidence.

^b^ Infection pressure is the number of infectious resident-days occurring in the window from 14 days prior (upper limit on one incubation period) to the outbreak identification date. The numeric value represents the odds ratio associated with a 1-unit increase in infection pressure.

^c^ Full follow-up defined as time from outbreak identification date until 14 days after the last detected case.

The 30-day SARS-CoV-2 incidence of infection (green) and mortality (orange) are shown as a function of the resident infection pressure on outbreak identification date (n = 632 outbreaks). Circle size is proportional to the number of outbreaks (n). Infection pressure is equal to the number of infectious resident-days at the time of outbreak identification, where residents were considered infectious from 2 days before onset to 8 days after onset. Outbreaks with infection pressure ≥ 3 were considered to be identified late, while outbreaks with infection pressure ≤ 2 were considered to be identified early.

### Additional analyses

The adjusted association between late outbreak identification and the incidence of infection at 30-days was similar in the first and second waves (adjusted OR_Wave 1_: 2.73; 95% CI: 1.88–3.97; adjusted OR_Wave 2_: 3.64; 95% CI: 2.11–6.29). When we limited the analysis to the second or subsequent outbreaks, impacts were similar to the overall effect (adjusted OR: 3.97; 95% CI: 2.90–5.43). When we limited the analysis to late-identified outbreaks, the continuous measure of infection pressure at the time of outbreak identification remained associated with increased risk (adjusted OR: 1.10; 95% CI: 1.08–1.13).

### Preventable cases through early outbreak identification

We conducted simulation analyses to determine the number of preventable cases and deaths if outbreak identification had occurred more rapidly. Starting from the observed outbreak identification delays, which yielded 7,698 cases and 2,219 deaths, if all outbreaks were identified early, rather than late, the simulation estimated that cases would have decreased to 5,455 (a reduction of 2,247 cases; 29.2%) and deaths would have decreased to 1,522 (a reduction of 697 deaths; 31.3%). When the same analysis was applied to Wave 1 and 2 separately, the simulation estimated that Wave 1 cases and deaths would have been reduced by 31.6% and 33.4%, respectively, while Wave 2 cases and deaths would have decreased by 23.2% and 24.3%, respectively.

## Discussion

This study examined the timeliness of outbreak identification and the subsequent secondary incidence of SARS-CoV-2 infections and deaths in LTC homes. We found that outbreaks identified late, when at least one resident was already symptomatic, evolved to be much larger outbreaks. Further, we identified a strong linear association between resident infection pressure at the time of outbreak identification and the overall size of outbreaks, with a 14% increased odds of infection among at-risk residents per additional infectious resident-day. We observed substantial improvements in the timeliness of outbreak identification between the first and second SARS-CoV-2 waves in Ontario, and estimated further reductions in the overall burden of SARS-CoV-2 that would have been possible with improvements to outbreak identification.

Prior studies have demonstrated initial underdetection of SARS-CoV-2 cases as a common feature during severe LTC home outbreaks. A case study of one of the first severe SARS-CoV-2 outbreaks from Ontario, Canada, reported that, at the time of outbreak declaration, 12 staff, two visitors and nine residents had symptoms [22]. One study from Fulton County, Georgia, during March–May 2020, conducted facility-wide testing within 1–5 days of an index resident SARS-CoV-2 case, showing that, on average 28% of residents and 7% of staff were already test-positive [23]. Our study developed a measure to determine the extent of an outbreak at the time an outbreak is identified, which can be used for future quality improvement initiatives. A paradoxical feature of this indicator is that we exclude staff infection pressure. Because of the important role of staff in propagating outbreaks, outbreaks without staff infection pressure are likely to represent outbreaks with lapses in staff case identification, and as such, inclusion could have weakened the predictiveness of our outbreak severity indicator.

Test turnaround times, frequency and sensitivity all have substantial impacts on outbreak detection [24]. Systematic delays in test turnaround times are apparent in LTC homes, particularly in for-profit homes and those with lower quality scores [25]. Actions to improve the timeliness of outbreak identification could reduce the severity of outbreaks. These could include, among others: (i) measures to promote earlier and more complete identification and testing of staff with symptoms of respiratory virus infection, such as through active screening and improved paid sick leave policies [26,27], (ii) testing of residents in proportion to population prevalence of infection [28] and (iii) strategies to reduce test turnaround times [29], such as point-of-care testing. At a health system level, systematic facility-level reporting of staff and resident respiratory virus symptoms and testing rates, and delays in outbreak identification, would facilitate setting of timeliness benchmarks (e.g. the 7-1-7 target [30]) and quality improvement through audit and feedback [31].

We observed substantial improvements in the quality and timeliness of outbreak identification between the first and second wave and concomitant reductions in outbreak size. These improvements were likely attributable to recognition of the importance of asymptomatic/pre-symptomatic transmission and atypical presentation in frail older adults, alongside increases in testing capacity and policy changes that occurred in Ontario [13]. Our results alongside a recent study [28], suggest increased test frequency could lead to earlier outbreak identification, and possibly to lower attack rates during outbreaks.

This study is subject to several limitations. Firstly, it was conducted before vaccine availability and before the emergence of new SARS-CoV-2 variants such as Alpha, Delta and the currently predominant Omicron variant, which may limit generalisability. Nevertheless, the insights from this study are still relevant for SARS-CoV-2 and other respiratory viruses since vaccine effectiveness for Omicron variants is low and wanes over time, many other seasonal respiratory infections lack effective vaccines, and because emergent infections are likely to lack effective vaccines as well. Thus, improving the timeliness of outbreak detection remains an important avenue for minimising the impacts of SARS-CoV-2 and other viral respiratory infections. Secondly, we lacked information on measures (other than transfer to the hospital) taken after cases were identified. However we were able to control for factors at the LTC home level including home crowding, which likely impacted the ability to isolate patients in rooms separately [9], and profit status, which is associated with staffing levels and quality [32]. Finally, our estimates of potential cases and deaths prevented through early outbreak identification may not have been achievable, since it may have been challenging to identify all outbreaks early in the first wave given the limited testing capacity.

## Conclusions

SARS-CoV-2 outbreaks in LTC homes, when identified late, evolved to be much larger than outbreaks identified early. The timeliness of outbreak identification can be used to predict the trajectory of an outbreak and to plan for increased staffing demands, infection control measures, and antiviral administration, with the goal of improving resident outcomes.

## Supplementary Data

135000 Supplement
