# Supplementary material for: Association between delayed outbreak identification and SARS-CoV-2 infection and mortality among long-term care home residents, Ontario, Canada, March to November 2020: a cohort study
Source: Euro Surveill. 2024 Oct 10;29(41):2300719. doi: 10.2807/1560-7917.ES.2024.29.41.2300719 (PMC11484918; doi:10.2807/1560-7917.ES.2024.29.41.2300719)
Supplement: Supplement [file 23-00719_BROWN_Supplement.pdf]

*This supplementary material is hosted by Eurosurveillance as supporting information alongside the article* Association between delayed outbreak identification and SARS-CoV-2 infection and mortality among long-term care home residents, Ontario, Canada, March to November 2020: a cohort study *, on behalf of the authors, who remain responsible for the accuracy and appropriateness of the content. The same standards for ethics, copyright, attributions and permissions as for the article apply. Supplements are not edited by Eurosurveillance and the journal is not responsible for the maintenance of any links or email addresses provided therein.*

Supplementary Table S1. Associations between risk factors and outbreak-associated SARS-CoV-2 infections and mortality in LTC homes, 30-day follow-up.

| | **30-day follow up** | |
|---|---|---|
| | **Incidence Adjusted* OR (95%CI)** | **Death Adjusted* OR (95%CI)** |
| Late outbreak identification (≥3 days of infection pressure) | 2.90 (2.04, 4.13) | 2.47 (1.77, 3.46) |
| **Resident characteristics** | | |
| Age ≥ 85 (per 10% increase) | 0.97 (0.78, 1.20) | 0.97 (0.80, 1.19) |
| Female (per 10% increase) | 1.11 (0.85, 1.46) | 1.06 (0.81, 1.38) |
| University education (per 10% increase) | 0.64 (0.45, 0.92) | 0.62 (0.45, 0.87) |
| Dementia (per 10% increase) | 1.00 (0.85, 1.19) | 0.99 (0.85, 1.16) |
| Comorbidities, mean** (per 0.1 increase) | 1.20 (0.97, 1.47) | 1.12 (0.92, 1.36) |
| ADL impairment scale (per 0.1 increase) | 1.11 (1.05, 1.17) | 1.10 (1.04, 1.15) |
| **Facility characteristics** | | |
| Facility size (per doubling) | 0.64 (0.50, 0.83) | 0.73 (0.58, 0.91) |
| Prior outbreaks | | |
| 0 | Reference | Reference |
| 1 | 1.79 (1.07, 2.99) | 1.83 (1.15, 2.92) |
| 2 | 0.73 (0.34, 1.54) | 0.80 (0.36, 1.77) |
| Profit-status | | |
| Municipal | Reference | Reference |
| Private non-profit | 1.15 (0.62, 2.13) | 1.35 (0.74, 2.46) |
| Private for profit | 1.53 (0.85, 2.75) | 1.70 (0.96, 3.00) |
| Crowding index >2 | 2.40 (1.61, 3.57) | 1.91 (1.32, 2.77) |
| **Region characteristics** | | |
| SARS-CoV-2 incidence on week of outbreak identification (cases per 100,000) *** | 1.26 (1.04, 1.53) | 1.25 (1.04, 1.52) |

Abbreviations: OR, odds ratio. * adjusted models included the following 12 covariates: the number of beds in the LTC home, LTC home proportion with age ≥85 years, proportion of female sex, proportion with university education, proportion with dementia, average number of comorbidities, average ADL impairment scale, crowding index > 2, profit status, prior outbreaks, outbreak identification date (spline with one knot for each month, not shown), and public health unit community SARS-CoV-2 incidence.
